# Next-century ocean acidification and warming both reduce calcification rate, but only acidification alters skeletal morphology of reef-building coral *Siderastrea siderea*

**DOI:** 10.1038/srep29613

**Published:** 2016-07-29

**Authors:** Kimmaree M. Horvath, Karl D. Castillo, Pualani Armstrong, Isaac T. Westfield, Travis Courtney, Justin B. Ries

**Affiliations:** 1 Department of Marine Sciences, University of North Carolina at Chapel Hill, Chapel Hill, North Carolina, 27599-3300 United States of America; 2 Department of Marine and Environmental Sciences, Northeastern University, Nahant, Massachusetts 01908, United States of America

## Abstract

Atmospheric *p*CO_2_ is predicted to rise from 400 to 900 ppm by year 2100, causing seawater temperature to increase by 1–4 °C and pH to decrease by 0.1–0.3. Sixty-day experiments were conducted to investigate the independent and combined impacts of acidification (*p*CO_2_ = 424–426, 888–940 ppm-v) and warming (T = 28, 32 °C) on calcification rate and skeletal morphology of the abundant and widespread Caribbean reef-building scleractinian coral *Siderastrea siderea*. Hierarchical linear mixed-effects modelling reveals that coral calcification rate was negatively impacted by both warming and acidification, with their combined effects yielding the most deleterious impact. Negative effects of warming (32 °C/424 ppm-v) and high-temperature acidification (32 °C/940 ppm-v) on calcification rate were apparent across both 30-day intervals of the experiment, while effects of low-temperature acidification (28 °C/888 ppm-v) were not apparent until the second 30-day interval—indicating delayed onset of acidification effects at lower temperatures. Notably, two measures of coral skeletal morphology–corallite height and corallite infilling–were negatively impacted by next-century acidification, but not by next-century warming. Therefore, while next-century ocean acidification and warming will reduce the rate at which corals build their skeletons, next-century acidification will also modify the morphology and, potentially, function of coral skeletons.

Atmospheric *p*CO_2_ has increased by over 30% from pre-industrial levels of *ca*. 270 to 280 ppm-v, causing surface seawater pH to decline by approximately 0.1^1^ and surface seawater temperature to increase by approximately 1 °C^2^. The Intergovernmental Panel on Climate Change^3^ predicts that atmospheric *p*CO_2_ will continue to increase to between 700 and 900 ppm-v by the end of the 21st century, which is predicted to cause surface seawater temperatures to increase by an additional 1 to 4 °C^3^,^4^ and sea surface pH to decrease by an additional 0.1 to 0.3^1^,^3^, thereby posing a significant threat for calcifying marine organisms^5^,^6^ and the ecosystems they comprise^7^,^8^.

The impact of ocean warming on calcification rates of scleractinian corals is relatively well established^9^,^10^. Rates generally increase up to a coral’s thermal optimum, which typically coincides with the coral’s average summertime seawater temperature^11^. Above this optimum, calcification rates begin to decline, in part due to bleaching^7^,^12^,^13^,^14^. The results of these controlled laboratory experiments are generally supported by coral-coring studies, which show that historical warming has caused declines in skeletal growth^9^,^15^,^16^,^17^,^18^, although some coring studies found that coral skeletal extension was either positively affected^10^,^19^,^20^,^21^ or unaffected^22^ by warming, with one study showing that a single species’ response to warming varied in both magnitude and direction across the reef (i.e., nearshore vs. backreef vs. forereef)^21^.

A large number of studies have investigated the impacts of ocean acidification on coral calcification^23^,^24^,^25^,^26^,^27^,^28^,^29^, with numerous reviews published on the subject^7^,^30^,^31^. These works reveal that the calcification response of scleractinian corals to ocean acidification varies widely amongst taxa^28^,^31^,^32^ and even within a taxon^25^,^31^,^33^. Many of these studies show that calcification rates of scleractinia decline relatively linearly with reductions in seawater pH^26^,^27^,^28^,^34^,^35^,^36^,^37^,^38^,^39^,^40^,^41^,^42^,^43^,^44^, while others have shown that scleractinia can exhibit no response, a threshold-negative response, or a parabolic response to CO_2_-induced ocean acidification^14^,^23^,^24^,^26^,^27^,^45^,^46^,^47^.

Since ocean warming and acidification are predicted to co-vary in coral reef systems over the coming centuries, it is important to constrain the combined effects of these two CO_2_-induced stressors on coral calcification. Several studies have shown that the negative effects of reduced seawater pH on coral calcification are exacerbated under elevated temperatures, suggesting a synergistic effect^6^,^39^,^45^,^48^. Other studies, however, report that elevated temperature has either no effect or a mitigating effect on the response of scleractinian corals to ocean acidification^27^,^32^,^33^,^38^,^47^,^49^.

Equally important to understanding the effect of ocean acidification and warming on coral calcification rate is the impact that these stressors will have on the structure of the coral skeleton. Few studies^44^,^50^ have quantified the impact of these individual stressors on coral skeletal morphology, and none has investigated the combined effects of warming and acidification on coral skeletal morphology. Cohen *et al*.^50^ found that new recruits of *Favium fragum* exposed to reduced aragonite saturation state (Ω_A_) exhibited decreased cross-sectional area (in plan-view) of the corallite structure and decreased aspect ratio of skeletal aragonite crystals. Likewise, Tambutté *et al*.^44^ found that adult specimens of *Stylophora pistillata* exhibited increased skeletal porosity and increased cross-sectional area (in plan-view) of the corallite cavity under highly elevated *p*CO_2_ of 2257 and 3793 μatm, but not under next-century *p*CO_2_ (856 μatm).

Here, we present the results of 60-day controlled laboratory experiments designed to assess the individual and combined effects of IPCC-predicted next-century ocean acidification (*p*CO_2_ = 424–426, 888–940 ppm-v) and warming (28, 32 °C) on the calcification rate (Table S1) and skeletal morphology (Table S2) of the tropical scleractinian coral *Siderastrea siderea*–an abundant and widespread reef-builder throughout Caribbean reef systems.

## Results

### Effect of temperature and *p*CO_2_ on calcification rate

**Figure 1:**
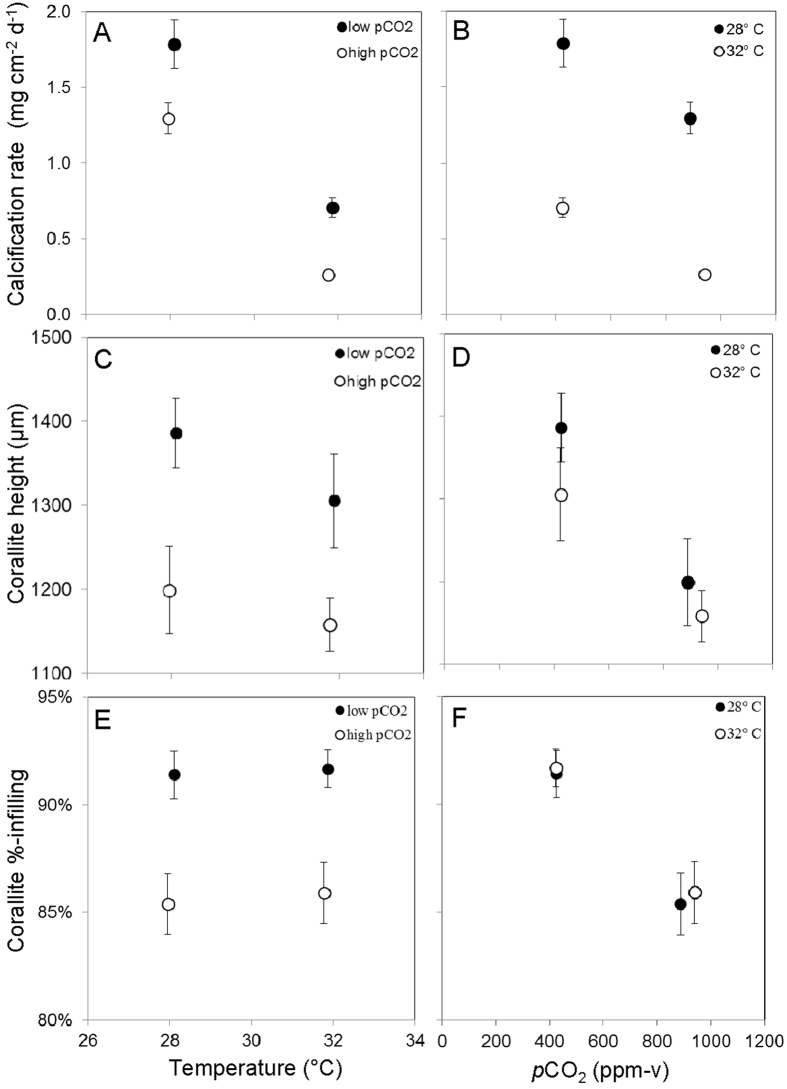
Effect of temperature and *p*CO_2_ on average calcification rates (**A,B**), corallite heights (**C,D**), and %-corallite infilling (**E,F**) of *S. siderea* corals reared in the four crossed temperature-*p*CO_2_ treatments (426 ppm-v/28.1 °C; 424 ppm-v/31.9 °C; 888 ppm-v/28.0 °C; 940 ppm-v/31.8 °C). Mixed effects modelling reveals that both *p*CO_2_ and temperature are significant predictors of coral calcification rate, while *p*CO_2_ alone is a significant predictor of corallite height and %-infilling, across the 60-day experiment. Bars show standard error.

Coral specimens under all temperature/*p*CO_2_ treatments exhibited net positive calcification (i.e., increase in total skeletal mass) throughout the experiment. However, net calcification rates declined with increasing temperature (28 °C vs. 32 °C) under both low and high *p*CO_2_ (Fig. 1A; Table S1) and with increasing *p*CO_2_ under both 28 and 32 °C (Fig. 1B; Table S1). Linear mixed effects modelling (Tables S3–4) that controlled for the random effects of tank and colony confirmed that temperature and *p*CO_2_ were significant (p < 0.05) predictors of calcification rate over the 60-day duration of the experiment (Table S5). The interactive effect of temperature and *p*CO_2_ was not a significant (p > 0.05) predictor of net calcification rate.

### Effect of temperature and *p*CO_2_ on bleaching

**Figure 2:**
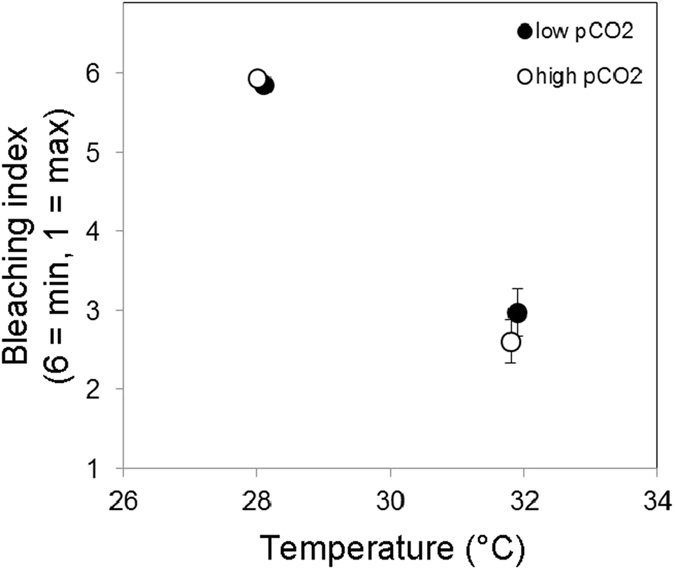
Bleaching index (‘1’ = total loss of pigment or maximum bleaching; ‘6’ = no loss of pigment or minimum bleaching) of *S. siderea* specimens reared in four crossed temperature-*p*CO_2_ treatments (426 ppm-v/28.1 °C; 424 ppm-v/31.9 °C; 888 ppm-v/28.0 °C; 940 ppm-v/31.8 °C). Bars show standard error.

Coral bleaching, defined here as a two-unit (or greater) decrease in the *Coral Watch Coral Health Chart* colorimetric bleaching index of the coral specimen between the beginning and end of the experiment^51^, was observed in the high temperature treatments (*ca*. 32 °C) at both 424 ppm-v and 940 ppm-v *p*CO_2_ (Figs 2, S1). Sixty-four percent of coral specimens in the high temperature/low *p*CO_2_ treatment exhibited decreased color saturation, with 25% of specimens exhibiting total loss of pigment. Seventy-five percent of coral specimens in the high temperature/high *p*CO_2_ treatment exhibited decreased saturation, with 28% of specimens exhibiting total loss of pigment. In the high temperature treatments, non-bleached coral specimens calcified faster than bleached specimens under both the low (0.89 ± 0.13 mg cm^−2^ d^−1^ vs. 0.60 ± 0.06 mg cm^−2^ d^−1^) and high *p*CO_2_ treatments (0.38 ± 0.06 mg cm^−2^ d^−1^ vs. 0.23 ± 0.02 mg cm^−2^ d^−1^; Fig. S2). No bleaching was observed in corals exposed to the low temperature treatment (*ca*. 28 °C) under either low or high *p*CO_2_ conditions (Figs 2, S1).

### Effect of exposure duration

**Figure 3:**
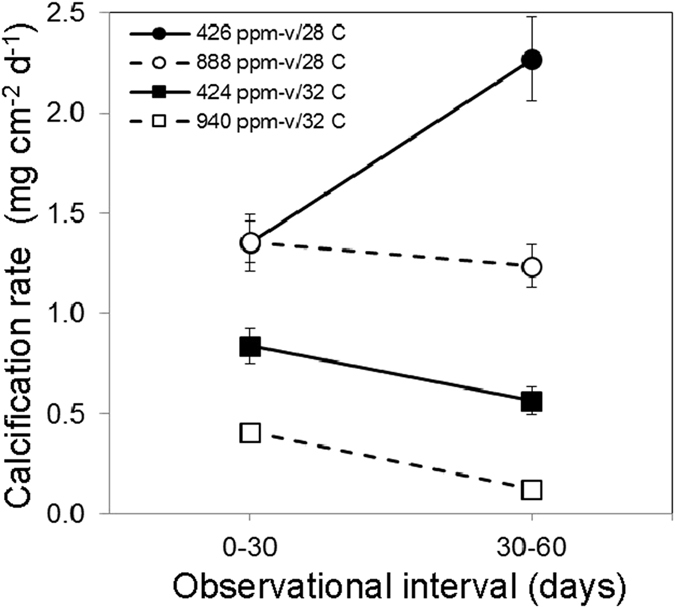
Average calcification rates for *S. siderea* corals reared under the control and elevated *p*CO_2_ ppm-v conditions for the 0–30 and 30–60 day observational intervals in four crossed temperature-*p*CO_2_ treatments (426 ppm-v/28.1 °C; 424 ppm-v/31.9 °C; 888 ppm-v/28.0 °C; 940 ppm-v/31.8 °C). Mixed effects modelling reveals that temperature only is a significant predictor of calcification rate across the 0–30 d observational interval, while both temperature and *p*CO_2_ are significant predictors across the 30–60 d and 0–60 d intervals. Bars show standard error.

For the 28 °C treatments, calcification rates in the 426 ppm-v treatment increased significantly (p < 0.05) between the 0–30 d and 30–60 d observational intervals, but were not significantly different (p > 0.05) between observational intervals in the 888 ppm-v treatment (Fig. 3). For the 32 °C treatments, calcification rates decreased significantly (p < 0.05) between the 0–30 d and 30–60 d intervals for both the low and high *p*CO_2_ treatments. Linear mixed effects modelling (Table S4) that controlled for the random effects of tank and colony revealed that temperature (p = 0.001), but not *p*CO_2_ (p > 0.05), was a significant predictor of calcification rate over the 0–30 d interval, while both temperature (p = 0.001) and *p*CO_2_ (p = 0.03) were significant predictors of calcification rate over the 30–60 d interval (Table S5).

### Effect of temperature and *p*CO_2_ on corallite height

Corallite height, defined here as the distance between the base and top of an individual corallite (the cavity onto which the individual coral polyp is anchored and retracts into when threatened; Fig. S3), was not significantly different (p > 0.05) between the 28 and 32 °C treatments under either low or high *p*CO_2_ conditions (Fig. 1C). However, corallite height was significantly (p < 0.05) lower in the high *p*CO_2_ treatment than in the low *p*CO_2_ treatment under both the 28 and 32 °C conditions (Fig. 1D). Linear mixed effects modelling (Table S4) that controlled for the random effects of tank and colony confirmed that temperature was not (p > 0.05) and that *p*CO_2_ was a significant (p = 0.003) predictor of corallite height over the 60-day duration of the experiment (Table S5). The interactive effect of temperature and *p*CO_2_ was not a significant (p > 0.05) predictor of corallite height.

### Effect of temperature and *p*CO_2_ on corallite infilling

There was no significant difference in average corallite infilling, defined as the percentage of corallite occupied by septal skeleton in plan view (Fig. S4), between the low and high temperature treatments under either low or high *p*CO_2_ conditions (Fig. 1E). However, average corallite infilling was significantly (p < 0.05) lower in the high *p*CO_2_ treatment than in the low *p*CO_2_ treatment under both the low and high temperature conditions (Fig. 1F). Linear mixed effects modelling (Table S4) that controlled for the random effects of tank and colony confirmed that temperature was not (p > 0.05) and that *p*CO_2_ was a significant (p = 0.003) predictor of corallite infilling over the 60-day duration of the experiment (Table S5). The interactive effect of temperature and *p*CO_2_ was not a significant (p > 0.05) predictor of corallite height.

**Figure 4:**
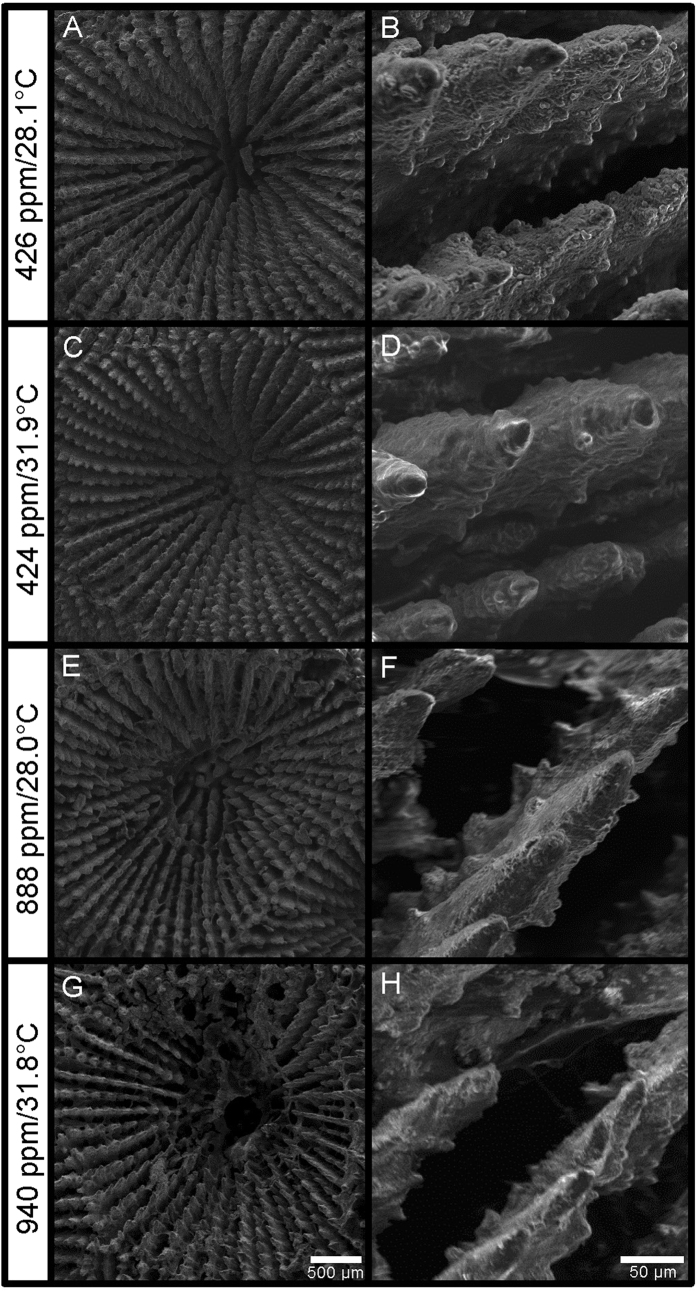
Secondary electron images of *S. siderea* corallites (left column) and septae (right column) produced under *p*CO_2_-temperature treatments of 426 ppm-v/28.1 °C (**A,B**), 424 ppm-v/31.9 °C (**C,D**), 888 ppm-v/28.0 °C (**E,F**), and 940 ppm-v/31.8 °C (**G,H**). Corallites are less infilled owing to thinner septae under conditions of elevated *p*CO_2_. Secondary electron images of the coral septae (right column) also reveal that elevated *p*CO_2_ decreases septal rugosity, apparently by reducing the prominence of the corals’ aragonite nucleation sites, their so-called centers-of-calcification.

A septal count revealed that the average number of septae per corallite (±SE) was not significantly different (p > 0.05) amongst treatments (28.1 °C/426 ppm-v = 46 ± 2; 31.9 °C/424 ppm-v = 46 ± 2; 28.0 °C/888 ppm-v = 45 ± 1; 31.8 °C/940 ppm-v = 47 ± 1), indicating that the observed decrease in corallite infilling with increasing *p*CO_2_ (Fig. 1F) resulted from CO_2_-induced septal narrowing, rather than from CO_2_-induced changes in the number of septae within each corallite. Secondary electron images (Fig. 4) of the corallites also support the assertion that the observed decrease in corallite infilling (Figs 1F and 4A,C,E,G) under elevated *p*CO_2_ results from narrowing of the coral septae (Fig. 4B,D,F,H). The secondary electron images of the coral septae also reveal that CO_2_-induced ocean acidification decreases septal rugosity, apparently by reducing the prominence of the corals’ aragonite nucleation sites, their so-called centers-of-calcification.

### Modelling calcification rate and skeletal morphology as a function of temperature and *p*CO_2_

Linear mixed effects modelling (Tables S4, S5) that controlled for the random effects of tank and colony identified the temperature-*p*CO_2_ model (additive) with random slopes for tank and colony (model 16 in Table S3) as the optimal model for predicting net calcification rate over the 0–60 d interval (interactive effects of temperature and *p*CO_2_ were not significant, p > 0.05).


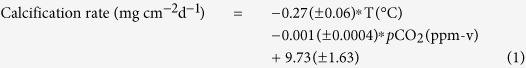


The temperature-only model (model 12 in Table S3) and the temperature-*p*CO_2_ model (additive, model 16 in Table S3), both with random slopes for tank and colony, were identified as the optimal models (Tables S4, S5) for predicting net calcification rate over the 0–30 d and 30–60 d observational intervals, respectively (interactive effects of temperature and *p*CO_2_ were not significant, p > 0.05).

The *p*CO_2_-only model with random slopes for tank and colony (model 20 in Table S3) was identified as the optimal model (Tables S4, S5) for predicting both corallite height and corallite infilling for the 0–60 d interval (neither additive nor interactive effects of temperature and *p*CO_2_ were significant, p > 0.05):


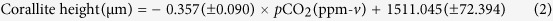



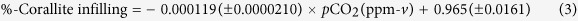


Linear mixed effects modelling across all treatments (Tables S4, S5) revealed that reef zone was not a significant (p > 0.05) predictor of calcification rate, corallite height, or corallite infilling, although nearshore corals calcified faster (p < 0.008) than forereef corals in the 888 ppm-v/28 °C treatment (Fig. S5).

## Discussion

### Effect of temperature on calcification rate

Results show that the calcification rate of the coral *S. siderea* declines at temperatures predicted for the next century (*ca*. 32 °C) for the Belize portion of the Meso-American Barrier Reef System (MBRS)^14^. These results are consistent with a prior study^14^ that observed a similar decline in calcification rate of *S. siderea* between temperature treatments of 28 and 32 °C under near-present day *p*CO_2_ conditions. At this temperature, the zooxanthellae that reside symbiotically within the coral’s tissues are expelled—a process known as bleaching^4^,^52^. Coral calcification appears to rely upon Ca^2+^-ATPase proton exchange mechanisms to increase the aragonite saturation of the corals’ calcifying fluid^53^,^54^,^55^, which may utilize energy provided by the coral’s symbiotic zooxanthellae in the form of translocated photosynthate. Thus, a decline in zooxanthellate abundance in the coral tissue due to bleaching should translate to a decline in available energy and, thus, a decline in calcification rate. Notably, coral bleaching resulted in a significant (p < 0.05) decrease in average calcification rates within the high temperature treatments (Fig. S2). In the 32 °C treatments, bleached corals calcified 32% slower than unbleached corals under near-present-day *p*CO_2_ conditions, and 40% slower under the high *p*CO_2_ conditions. Thus, much of the observed decline in coral calcification rate under the high-temperature treatments (Fig. 1A) appears to be linked to the bleaching that occurred under these conditions (Figs 2, S2). It should also be noted that the prescribed temperature difference between treatments (28 vs. 32 °C) caused an approximate 1 unit difference in Ω_A_ (higher T = higher Ω_A_) under both low and high *p*CO_2_ conditions—a relationship that also exists in natural reef settings. It is therefore possible that the negative impact of elevated temperature on calcification rate was partially mitigated by a small temperature-induced increase in Ω_A_.

### Effect of *p*CO_2_ on calcification rate

Results suggest that calcification within the scleractinian coral *S. siderea* will be impaired by CO_2_-induced ocean acidification that is predicted for year 2100^3^. These results are consistent with some studies investigating the impact of CO_2_-induced ocean acidification on tropical corals^36^,^56^, but contrast other studies on tropical^24^,^45^, temperate^23^, and cold water^57^ scleractinian corals that found that calcification rates were not impaired by CO_2_-induced acidification of comparable magnitude. Collectively, these results support the assertion that ocean acidification poses a substantial threat to scleractinian corals, but that their specific response to this environmental stressor is highly variable and complex.

A prior study^14^ investigating the effects of ocean acidification on *S. siderea* found no statistically significant change in calcification rate between *p*CO_2_ of 477 and 604 ppm-v (28 °C), but observed a significant decline in net calcification rate between *p*CO_2_ of 604 and 2553 ppm-v (28 °C) that was of a similar magnitude to the decline observed in the present study between *p*CO_2_ of 424 and 888 ppm-v (28 °C). Overlapping these two sets of results suggests that *S. siderea* exhibits a substantial, threshold decline in calcification rate between *p*CO_2_ of approximately 600 and 900 ppm-v, with ocean acidification having little impact on net calcification rate of this species outside of that *p*CO_2_ range.

### Combined effects of temperature and *p*CO_2_ on calcification rate

An important objective of the present study was to investigate the *combined* effects of ocean warming and acidification on the calcification rate and skeletal morphology of a tropical reef-building coral, as these stressors are predicted to co-vary in reef systems over the foreseeable future^3^. Notably, of the various combinations of stressors, it was the high-temperature/high-*p*CO_2_ treatment that yielded the greatest percentage decrease in rate of coral calcification relative to the control (85% decline). Furthermore, this treatment was the only one to yield negative calcification rates (i.e., net dissolution) for some specimens (Table S1), although the mean net calcification rate for that treatment was positive. However, the observation that the interactive effect of *p*CO_2_ and temperature on calcification rate was not significant (p > 0.05), while the additive effect of these stressors was significant (p < 0.05), suggest that the combined impacts of these stressors on the calcification rate and skeletal morphology of this species are not, in the strict definition of the word, synergistic.

Predictive equations generated from the mixed effects modelling show that for every 1 °C rise in temperature, an approximately 270 ppm-v rise in atmospheric *p*CO_2_ is required to produce an equivalent decline in net coral calcification rate. Thus, the investigated change in temperature (4 °C increase) exerts a relatively greater effect than the investigated change in *p*CO_2_ (500 ppm-v increase) on net calcification rate of this coral species. This is supported by the observation that the linear mixed effect modelling identified temperature as a significant (p < 0.05) stand-alone predictor of calcification rate over the 60-day duration of the experiment, while *p*CO_2_ was only a significant predictor of calcification rate when combined with temperature in the additive model (Table S5, S4). Although these results are consistent with prior work^14^ showing that the isolated effects of predicted next-century warming on *S. siderea* calcification are more severe than the isolated effects of predicted next-century acidification, the present study shows that it is the combination of these stressors that yields the most deleterious outcome for this species.

### Effect of exposure duration

Buoyant weights obtained at 30-day intervals throughout the experiment revealed that, at 32 °C, the deleterious effect of elevated *p*CO_2_ on calcification rate was observed in both the first and second observational intervals (0–30 d, 30–60 d), while at 28 °C it was not observed until the second observational interval (Fig. 3; Table S5). This delayed response at 28 °C may result from a more gradual depletion of the coral’s energy reserves^14^,^50^,^58^,^59^, culminating during the second observational interval of the experiment. In contrast, the potentially more immediate depletion of energy reserves in the 32 °C treatment may have led to the correspondingly more immediate manifestation of the negative impacts of acidification within that treatment.

The process of coral skeletal formation appears to require the removal of protons from a coral’s calcifying fluid, which requires energy^40^,^50^,^53^,^54^,^60^,^61^. Removing protons from a calcifying fluid that is surrounded by seawater of higher proton concentrations (i.e., lower pH) requires transporting protons across a stronger proton gradient, which should, in turn, require more energy^54^. It is therefore possible that the delayed impact of elevated *p*CO_2_ on calcification rates of *S. siderea* maintained under the low temperature condition (28 °C) is attributable to the progressive depletion of energy reserves amidst the increased energetic demands of transporting protons across a stronger proton gradient under higher *p*CO_2_-conditions.

Conversely, it is possible that the deleterious effects of elevated *p*CO_2_ on calcification rate were evident in both the first and second observational intervals of the high temperature treatments (32 °C) because the combined stress of warming and acidification caused the coral’s energy reserves to become depleted in fewer than 30 days (i.e., during the first observational interval), with further declines observed during the second observational interval (Fig. 3). This depletion of energy reserves may have been exacerbated in the higher temperature treatments by bleaching, which would have reduced the coral’s production of photosynthate.

The delayed effects of seawater warming and acidification on coral calcification rate, possibly resulting from progressive depletion of energy reserves, may explain some of the variation in magnitude, and even direction, of calcification responses to warming and acidification observed in prior experiments on tropical corals that were conducted over different durations^2^,^5^,^28^,^34^,^35^,^38^,^40^,^41^. These results underscore the importance of conducting experiments investigating the effects of environmental stressors on coral calcification over a range of timescales, in order to assess short-, mid-, and long-term responses.

### Effect of temperature and *p*CO_2_ on corallite geometry

The corallite (Figs 4, S3) is a critical component of the coral skeleton because it defines the protective cavity that the coral polyp inhabits and retreats into when threatened. The corallite is partitioned by vertical plates (septae) radiating from the center, which the polyp uses to anchor itself into the corallite. Both height and infilling of the corallite were significantly lower (p < 0.05) in the high *p*CO_2_ treatments than in the low *p*CO_2_ treatments, under both the 28 and 32 °C conditions (Figs 1D,F, 4). Although a trend towards lower corallite height under the higher temperature conditions was noted (Fig. 1C), it was not statistically significant (p > 0.05). Hierarchical mixed effects modelling confirmed that *p*CO_2_ was the only statistically significant predictor of corallite height and infilling (Table S5).

The observed reductions in corallite height and infilling under elevated *p*CO_2_ (Figs 1D,F, 4) are consistent with the observed reductions in net calcification rate under elevated *p*CO_2_ (Fig. 1B) and may reveal one of the pathways by which CO_2_-induced ocean acidification leads to reduced rates of calcification within this coral species. Ultimately, these observed impacts of elevated *p*CO_2_ on coral skeletal morphology may result from a decrease in aspect ratio (i.e., length:width) of the coral’s individual aragonite crystals formed under lower Ω_A_^26^,^50^ (i.e., conditions closer to equilibrium favor lower aragonite crystal aspect ratios).

The observed reduction in corallite infilling under elevated *p*CO_2_ is consistent with the results of an 8-day experiment conducted at 25 °C by Cohen *et al*.^50^ that found that the cross-sectional skeletal area (in plan view) of new recruits of *Favia fragum* was approximately 25% lower when grown under next-century Ω_A_, although a direct comparison with the results of the present study is precluded by the studies’ contrasting methods (acid addition vs. *p*CO_2_ manipulation). The results of the present study on *S. siderea*, however, are not consistent with the results of Tambutté *et al*.^44^, which found that next-century acidification (*p*CO_2_ of 856 μatm vs. 538 μatm control) did not cause changes in the skeletal morphology of *Stylophora pistillata*, although changes were observed under much higher *p*CO_2_ (2257, 3793 μatm).

These results suggest that atmospheric *p*CO_2_ predicted for the next century will alter the skeletal morphology of *S. siderea* by reducing corallite height and extent of corallite infilling (Figs 1D,F, 4). CO_2_-induced reductions in corallite height (Fig. 1D) reduce the volume of the corallite into which the coral polyp can retreat when threatened, while CO_2_-induced reductions in septal width and rugosity (Figs 1F, 4) may make it harder for the polyp to anchor itself within the corallite—both potentially increasing the polyp’s vulnerability to predation. CO_2_-induced reductions in corallite infilling and septal width may also reduce the biomechanical strength of the corallite, thereby impairing the corallite’s ability to withstand mechanical impact and/or abrasion from high-force events such as storms, tsunamis, boat groundings, and parrotfish grazing.

### Implications of study results

The results of this experiment show that although both warming and acidification negatively impact calcification rates of the abundant and widespread Caribbean coral *S. siderea*, it is their combined effect that yields the most deleterious impacts. Corallite height and corallite infilling, two relatively easily measured and ecophysiologically relevant parameters for quantifying the impact of environmental stress on coral skeletal morphology, were found to be negatively impacted by acidification, but not by warming. Collectively, these results suggest that *S. siderea* will not only grow more slowly in warmer, more acidic oceans predicted for the future, but will also produce a skeleton of modified structure and, potentially, function.

## Methods

### Specimen collection and acclimation

Eighteen colonies of *S. siderea* were collected via SCUBA along the MBRS, approximately 40 km east of the Belize coast, within the Sapodilla Cayes Marine Reserve (16° 06′ 09′′ N/88° 16′ 20′′ W and 16° 07′ 00′′ N/88° 16′ 01′′ W) in June 2011, in accordance with local, federal, and international regulations. *Siderastrea siderea* colonies (20–30 year old) were selected randomly from 4–5 m deep waters of the MBRS. Colonies were collected at a minimum of 0.5 km apart to maximize genotypic variability. Colonies were wrapped in seawater-moistened paper towels and transported by airplane to the University of North Carolina at Chapel Hill, where they were cut into *ca*. 2 cm × 2 cm fragments with a seawater-cooled petrographic trim saw. Individual coral fragments were affixed with cyanoacrylate to acrylic slides and given unique identification. Fragments were then acclimated to laboratory conditions for 30 days and then to experimental conditions for an additional 14 days. Seawater temperature and *p*CO_2_ were incrementally adjusted to treatment levels leading up to the final 14-day acclimation period in order to minimize shock to the corals.

### Experimental conditions

Four experimental treatments of two seawater temperatures (*ca*. 28, 32 °C) crossed with two *p*CO_2_ levels (*ca*. 424–426 ppm-v, 888–940 ppm-v) were established. The two temperatures were chosen to coincide with the current average annual temperature on the MBRS in Belize (*ca*. 28 °C) and the IPCC (2014) worst-case scenario temperature increase of 4 °C (*ca*. 32 °C). The two *p*CO_2_ treatments were selected to represent near present day conditions (*ca*. 424–426 ppm-v) and a predicted end-of-century level (*ca*. 888–940 ppm-v). Each of the four treatments was maintained in triplicate 38 L glass aquaria (12 aquaria total). Twelve similarly sized *S. siderea* fragments sourced from the same suite of coral colonies were transferred to each of the 12 aquaria (144 total fragments).

Experimental seawater was prepared from deionized water and *Instant Ocean Sea Salt* at a salinity (±SE) of 35.10 ± 0.02. Seventy-percent water changes were performed approximately every 10 days with *ca*. 35 salinity artificial seawater, with deionized water added as needed to replenish water lost through evaporation. Seawater in each aquarium was continuously filtered with activated charcoal and polyester fleece at a rate of 757 L/h. Water circulation within each tank was enhanced with a 400 L/h powerhead (*Maxi-Jet* 400) attached to each aquarium wall. Aquaria were covered with plexiglass lids and cellophane wrap to minimize evaporative water loss and gas exchange with the room air.

Aquaria were illuminated with a timer-controlled 4-stage daily light cycle in order to mimic reef-conditions: 12 hours dark (no light); 1 hour dawn (ultra-actinic-blue light); 10 hours daylight (ultra-actinic-blue light + 96 Watt 10,000 K white light + 32 Watt 6500 K fluorescent light); 1 hour dusk (ultra-actinic-blue light). The maximum photosynthetically active radiation (PAR) of the daily light cycles was *ca*. 250 μmol photons m^−2^ s^−1^.

Each coral fragment was hand-fed approximately 1.25 g (wet-weight) *Artemia* sp. twice weekly. Seawater temperatures (*ca*. 28 °C and 32 °C) were maintained with 50 W submersible aquarium heaters, which were calibrated with NIST-traceable glass thermometers.

*Aalborg* digital solenoid-valve mass flow controllers were used to mix compressed CO_2_ gas with compressed air to achieve gas mixtures of the desired *p*CO_2_: 426 ± 11 and 888 ± 14 ppm-v at *ca*. 28 °C; 424 ± 10 and 940 ± 10 ppm-v at *ca*. 32 °C (Table 1). These gas mixtures were sparged into the seawater treatments via micro-porous ceramic gas bubblers.

### Measurement and calculation of seawater parameters

Seawater temperature, salinity, and pH were measured three times per week throughout the duration of the experiment (Table 1). Temperature was determined with a NIST-calibrated partial-immersion organic-filled glass thermometer. Salinity was determined with a *YSI 3200* conductivity meter outfitted with a *YSI 3440* conductivity cell (K = 10), which was calibrated with seawater standards of known salinity supplied by the laboratory of Prof. A. Dickson of Scripps Institution of Oceanography. Seawater pH was determined with an *Orion* benchtop pH meter and an *Orion* Ross pH electrode calibrated with 7.00 and 10.01 certified NBS buffers traceable to NIST standard reference material (for slope of the calibration curve) and with seawater standards of known pH provided by the laboratory of Prof. A. Dickson (for y-intercept of the calibration curve). Approximately 250 ml seawater samples were collected weekly from experimental aquaria in accordance with published best practices^62^ and analyzed for dissolved inorganic carbon (DIC, via coulometry) and total alkalinity (TA, via closed-cell potentiometric titration) using a *MARIANDA* corporation *VINDTA 3 C* (Table 1). Seawater *p*CO_2_, pH, carbonate ion concentration ([CO_3_^2−^]), bicarbonate ion concentration ([HCO_3_^−^]), aqueous CO_2_, and Ω_A_ were calculated with the program CO2SYS^63^, using Roy *et al*.^64^ values for the K_1_ and K_2_ carbonic acid constants, the Mucci^65^ value for the stoichiometric aragonite solubility product, and an atmospheric pressure of 1.015 atm (Table 1).

### Quantification of coral bleaching

Coral specimens were photographed with the *Coral Watch Coral Health Chart* (University of Queensland) colorimetric reference card at the start (0 d), midpoint (30 d), and completion (60 d) of the experiment with a *Canon* digital camera mounted to a stand, using identical illumination, camera settings, and working distances. The color saturation of the coral specimens’ tissue was ranked on a 6-point scale via visual comparison with the colorimetric reference card (6 = maximum color saturation; 1 = no pigment). Bleaching was considered to have occurred when a decrease in color saturation of two or more units was observed between the beginning and end of the experiment^51^,^66^.

### Quantification of net coral calcification rate

Net rates of coral calcification (Table S1) were estimated from buoyant weights determined at the beginning, middle, and end of the experiment. The buoyant weight-dry weight relationship for the coral *S. siderea* was empirically derived by plotting final dry weights against final buoyant weights of coral specimens separately for the low- and high-*p*CO_2_ treatments (Fig. S6). Buoyant weight vs. dry weight regressions were highly linearly correlated for corals from both *p*CO_2_ treatments (R^2^ = 0.94 for the low-*p*CO_2_ treatment; R^2^ = 0.99 for the high-*p*CO_2_ treatment), indicating that linear equations could be used to convert buoyant weight to dry weight for the purpose of estimating net calcification rates:


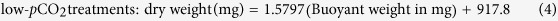



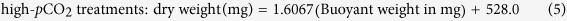


Net calcification rates were normalized to coral fragment surface area and observational interval and expressed as mg (dry weight) cm^−2^ d^−1^ (Table S1).

### Assessment of corallite morphology

Coral specimens (n = 12–22) were selected from each of the four treatments for morphological analysis via stereomicroscopy and scanning electron microscopy. Specimens were equitably sourced from the eleven colonies to control for intercolonial variation and submerged for three hours in 8.25% sodium hypochlorite solution to remove organic residue that could obscure corallite morphology when viewed under magnification. Samples were then rinsed with 95% ethanol and air-dried.

Corallite height (Fig. S3; Table S2) was determined via stereomicroscopy (*Nikon SMZ1500*) as the difference in vertical position of the microscope’s z-stage (calibrated to 0.1 μm with a certified micrometer) when the base versus the top of the corallite was in focus. Specimens with predominantly flat upper surfaces were selected to ensure that vertical distances between the base and top of the corallite approximated true corallite height. A minimum of 3 corallites per specimen, 5 specimens per replicate, and 16 specimens per treatment were needed to obtain a normal distribution of data.

Percent-infilling of the corallite (Fig. S4; Table S2) was quantified via 8-bit gray-scale image analysis (*ImageJ*) of fully-focused top-down images of individual corallites. Fully focused images of the corallites were obtained using a *Nikon SMZ1500* microscope fit with an automated z-stage system, a *Nikon Digital Sight DS-Ri1* camera, and *NIS Elements* image processing software. Images were captured using ‘auto exposure’ and ‘auto white balance’ acquisition settings, with stage and room illumination held constant. The focused portions of the separate z-stacked images were aligned and merged into a single fully focused image (Fig. S4a) using the imaging software. This fully focused image was then imported into the image processing software program *ImageJ* and converted to an 8-bit grayscale photo (Fig. S4b). The contrast of this image was increased by 30% (Fig. S4c) to further distinguish positive space (i.e., corallite infilling) from negative space (i.e., lack of corallite infilling). The perimeter of the corallite of interest was manually cropped from the larger image (Fig. S4d), and the number of septae per corallite was recorded. The ‘histogram tool’ of *ImageJ* was then applied to the final image to rank the intensity of each pixel on a scale of 0 (black) to 256 (white). A pixel intensity (PI) of 20 was selected as the divide between negative space (PI ≤ 20; lack of corallite infilling) and positive space (PI > 20; corallite infilling), as PI > 20 accurately captured corallite infilling while excluding darker pigmentation resulting from void space. The program *R* was used to tally pixel intensity distribution, with percent corallite infilling calculated as the percentage of pixels with PI > 20. A minimum of 1 corallite per specimen, 2 specimens per replicate tank, and 12 specimens per treatment were needed to obtain a normal distribution of data.

Secondary electron images of coral specimens from each of the four treatments were obtained with a tungsten-filament variable pressure *Tescan Vega 3 LMU* scanning electron microscope (accelerating voltage = 20 kV) under high-vacuum at 50- to 200-times magnification.

### Statistical analyses

Hierarchical linear mixed-effects models were utilized to fit the two-way factorial experiment with split-plot design to assess the additive and interactive effects of *p*CO_2_, temperature, and reef-zone on the calcification rate, corallite height, and corallite infilling of *S. siderea* over the 60-day experiment (Tables S3, S4, S5). Tanks represent plots, temperature and *p*CO_2_ represent whole-plot treatments, and coral colonies and reef-zones represent split-plot treatments. Random effects at the colony level were employed to control for potential genotypic effects, and random effects at the tank level were employed to control for potential ‘tank-effects’. The random effects of tank and colony were crossed and nested within the fixed effects of *p*CO_2_, temperature, and reef-zone. Five model-types with different combinations of fixed effects to evaluate the independent, additive, and interactive effects of *p*CO_2_, temperature, and reef-zone were examined with the crossed random effects of tank and colony randomized at different levels of the model, for a total of 20 models (Table S3).

All linear mixed-effects models were estimated using the *lme4* package of *R* 3.0.2 (Table S4). Restricted maximum likelihood (REML) was used to fit each model and calculate unbiased estimates of parameter variance and standard error. The *AFEX*-package in *R* was used to obtain parameter p-values (Table S5) for the linear mixed-effects models, using the Kenward-Roger approximation for degrees-of-freedom. Optimal models (Tables S4, S5) were identified as those yielding the greatest number of significant (p < 0.05) fixed effects, with random effects assigned by AIC. The reported variance of the random effects (tank, colony) is proportional to the relative magnitude of their impacts on the dependent variable (calcification rate, corallite height, corallite infilling).

## Additional Information

**How to cite this article**: Horvath, K. M. *et al*. Next-century ocean acidification and warming both reduce calcification rate, but only acidification alters skeletal morphology of reef-building coral *Siderastrea siderea*. *Sci. Rep*. **6**, 29613; doi: 10.1038/srep29613 (2016).

## Supplementary Material

Supplementary Information

## Figures and Tables

**Table 1:** Summary of average calculated and measured parameters for the experimental treatments.

CALCULATED PARAMETERS					
*p*CO_2 (gas-e)_	(ppm-v)	424	426	940	888
	SE	10	11	10	14
	Range	349–537	334–522	824–1059	730–1018
	n	24	22	27	26
pH_C_		8.09	8.10	7.80	7.77
	SE	0.01	0.01	0.01	0.01
	Range	7.93–8.16	7.96–8.19	7.76–7.85	7.69–7.85
	n	24	22	27	26
[CO_3_^2−^]	(μM)	413	363	233	170
	SE	13	12	4	5
	Range	249–470	239–435	204–268	130–218
	n	24	22	27	26
[HCO_3_^−^]	(μM)	2069	2104	2338	2090
	SE	24	28	19	27
	Range	1797–2232	1873–2317	2240–2542	1857–2329
	n	24	22	27	26
[CO_2_] _(SW)_	(μM)	10.2	11.2	22.6	23.4
	SE	0.2	0.3	0.2	0.4
	Range	8.4–12.7	8.8–13.6	19.9–25.4	19.1–27.0
	n	24	22	27	26
Ω_A_		6.8	5.8	3.8	2.7
	SE	0.2	0.2	0.1	0.1
	Range	4.1–7.7	3.8–7.0	3.4–4.4	2.1–3.5
	n	24	22	27	26
					
**MEASURED PARAMETERS**					
Sal		35.15	35.30	34.88	35.04
	SE	0.05	0.05	0.04	0.04
	Range	34.50–36.50	34.60–36.70	34.00–35.50	34.30–35.80
	n	81	81	78	78
T	(°C)	31.9	28.1	31.8	28.0
	SE	0.1	0.1	0.1	0.1
	Range	31.0–32.7	27.5–29.1	31.1–32.1	27.7–28.3
	n	81	81	78	78
pH_M_		8.14	8.08	7.89	7.82
	SE	0.01	0.01	0.01	0.01
	Range	8.00–8.28	8.01–8.18	7.75–8.21	7.62–8.18
	n	81	81	78	78
TA	(μM)	3024	2948	2878	2493
	SE	51	50	26	36
	Range	2404–3217	2459–3239	2723–3118	2190–2821
	n	24	22	27	26
DIC	(μM)	2492	2478	2593	2283
	SE	36	37	22	31
	Range	2065–2672	2133–2723	2470–2814	2017–2563
	n	24	22	27	26

Abbreviations: ‘*p*CO_2 (gas-e)_’ = *p*CO_2_ of the mixed gas in equilibrium with the experimental seawaters; ‘pH_C_’ = calculated pH; ‘[CO_3_^2−^]’ = carbonate ion concentration; ‘[HCO_3_^−^]’ = bicarbonate ion concentration; [CO_2_] _(SW)_; ‘Ω_A_’ = aragonite saturation state; ‘Sal’ = salinity; ‘T’ = temperature, ‘pH_M_’ = measured pH; ‘TA’ = total alkalinity, ‘DIC’ = dissolved inorganic carbon; ‘SE’ = standard error of the mean; ‘n’ = number of observations.
