# Supplementary material for: Next-century ocean acidification and warming both reduce calcification rate, but only acidification alters skeletal morphology of reef-building coral Siderastrea siderea
Source: Sci Rep. 2016 Jul 29;6:29613. doi: 10.1038/srep29613 (PMC4965865; doi:10.1038/srep29613)
Supplement: Supplementary Information [file srep29613-s1.pdf]

Supplementary Information

Next-century ocean acidification and warming both reduce calcification rate, but only acidification (not warming) alters skeletal morphology of reef-building coral *Siderastrea siderea*

KIMMAREE M. HORVATH, KARL D. CASTILLO, PUALANI ARMSTRONG, ISAAC T. WESTFIELD, TRAVIS COURTNEY, and JUSTIN B. RIES

Supplementary figures S1 – S6 and tables S1 – S5

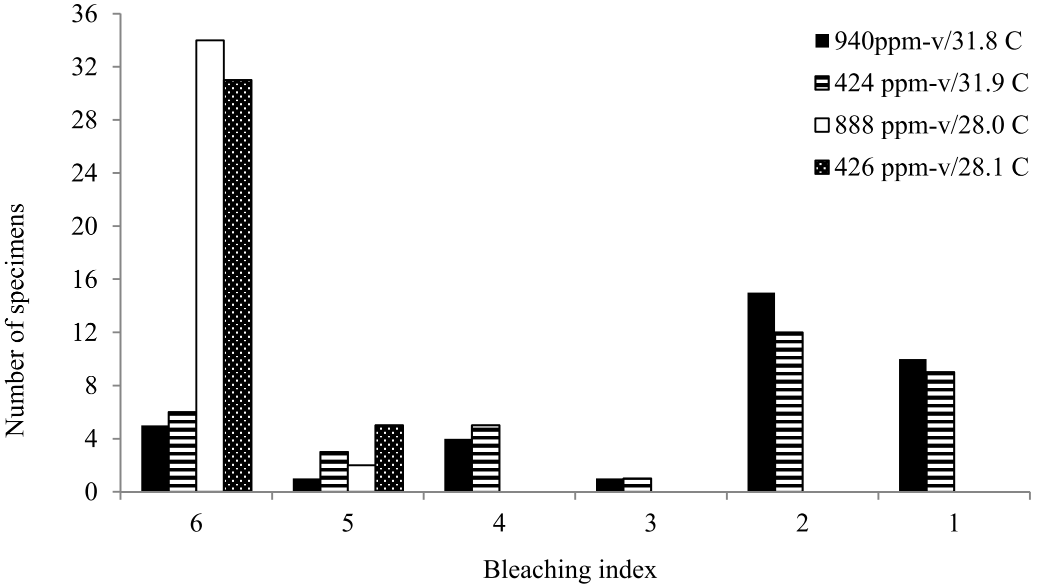

Figure S1. Histogram distribution of 'bleaching index' ('1' = total loss of pigment; '6' = maximum color saturation; *Coral Watch Coral Health Chart*) for *S. siderea* specimens after 60 days of exposure to the four temperature-pCO_2 treatments.

(a)

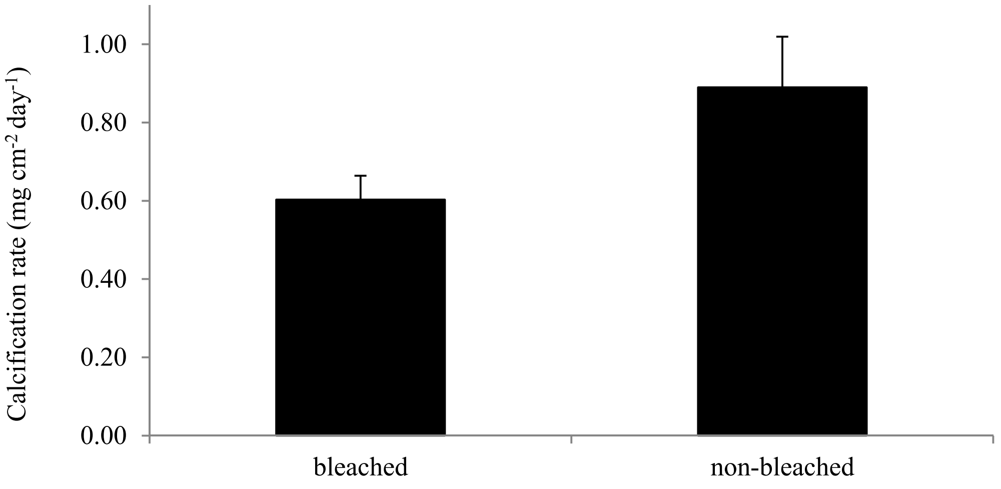

(b)

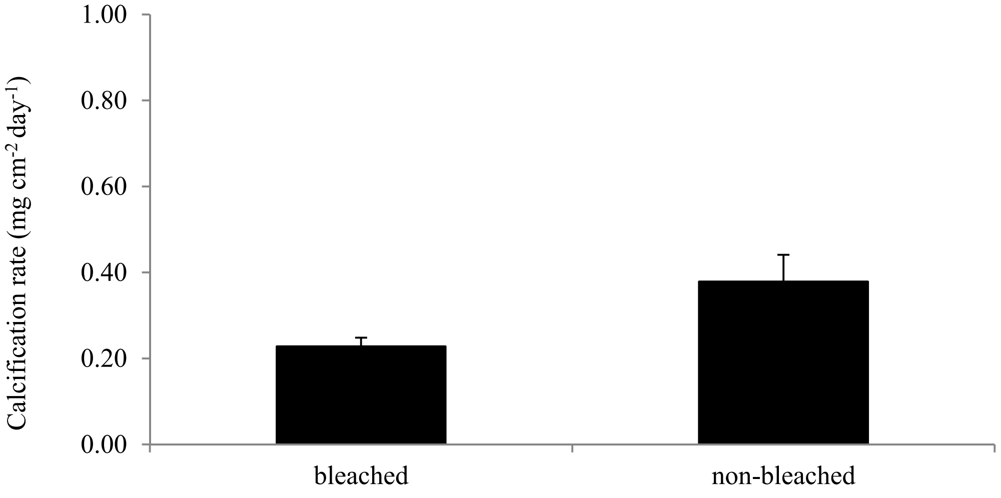

Figure S2. Average calcification rate for bleached vs. non-bleached specimens of *S. siderea* in 31.9 °C-424 ppm-v (a) and 31.8 °C-940 ppm-v (b) treatments. 'Bleaching' is defined as a two or more unit decrease in bleaching index (1 – 6 scale, *Coral Watch Coral Health Chart*) for a coral specimen between the beginning and end of the experiment. Black vertical bars show standard error.

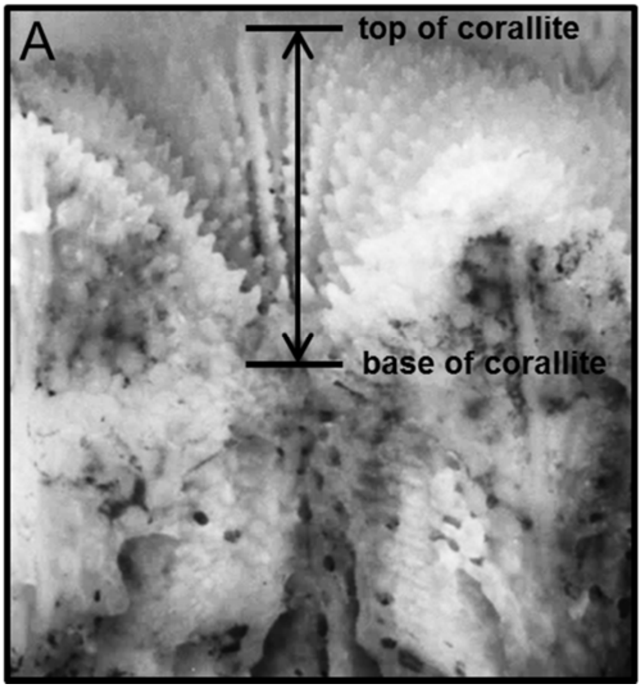

Figure S3. Cross-sectional photomicrograph of the corallite structure, showing location of corallite height measurement (arrow) obtained in the present study via stereomicroscopy. Arrow is *ca.* 1200 – 1400 μm.

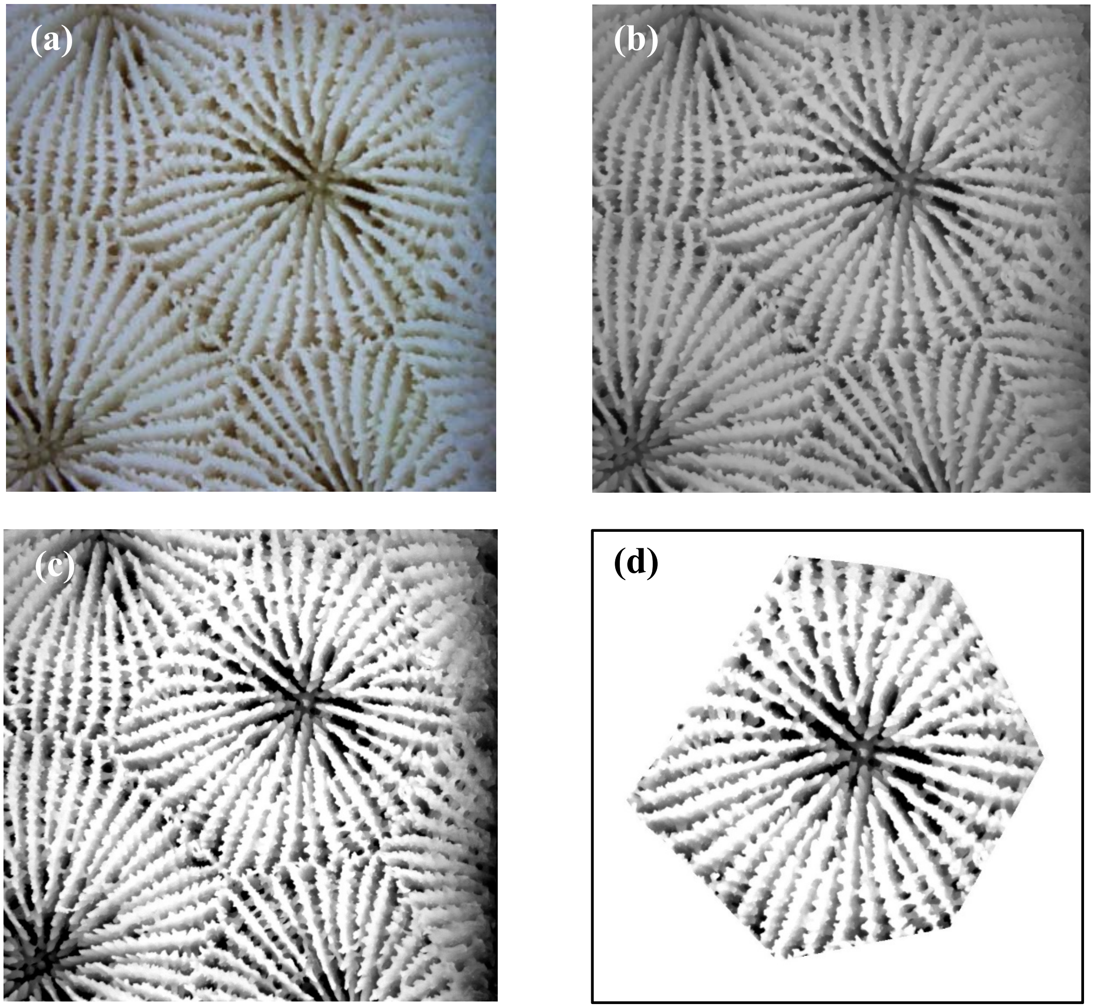

Figure S4. Progressive manipulation of photomicrographs of *S. siderea* corallite for quantification of %-infilling: (a) original aligned and fully-focused 32-bit color image; (b) 32-bit color image converted to 8-bit grayscale image; (c) image contrast increased by 30%; and (d) individual corallite cropped for analysis.

(a)

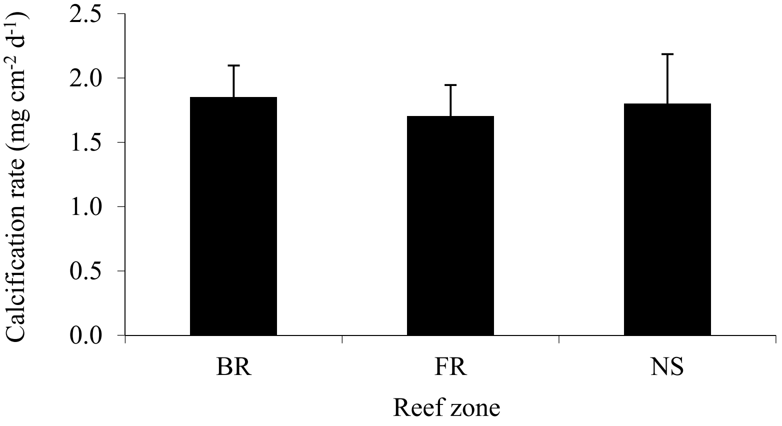
Calcification rate (mg cm-2 d-1)
2.5
2.0
1.5
1.0
0.5
0.0
BR
FR
NS
Reef zone

(b)

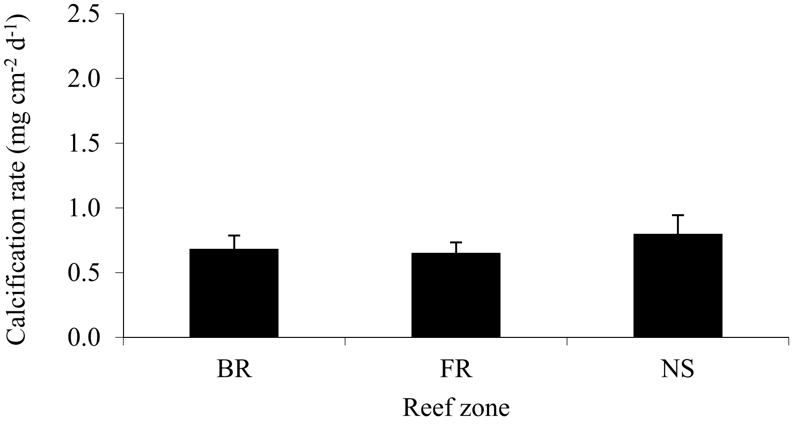
Calcification rate (mg cm-2 d-1)
2.5
2.0
1.5
1.0
0.5
0.0
BR
FR
NS
Reef zone

(c)

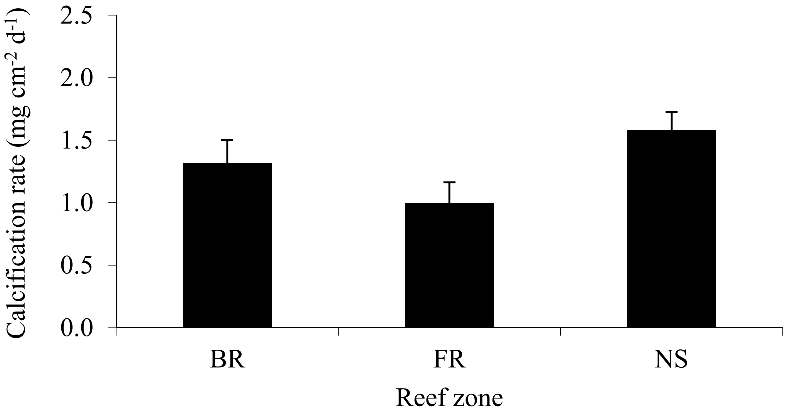
Calcification rate (mg cm-2 d-1)
2.5
2.0
1.5
1.0
0.5
0.0
BR
FR
NS
Reef zone

(d)

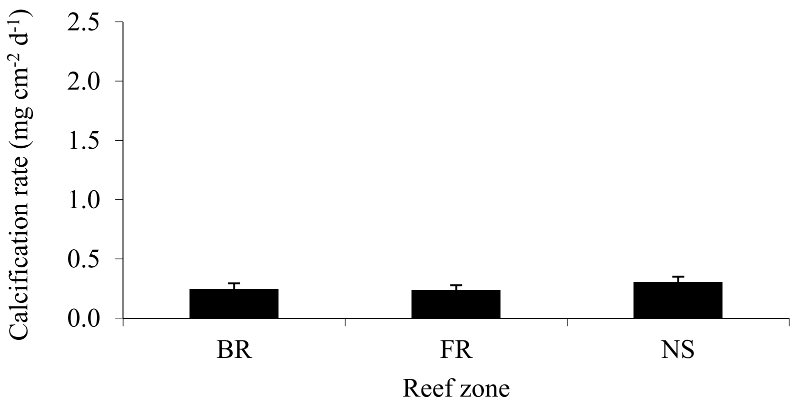

Figure S5. Calcification rates of *S. siderea* coral specimens from different reef zones ('BR' = backreef; 'FR' = forereef; 'NS' = nearshore) within the four temperature-pCO_2 treatments: (a) 426 ppmv-28.1 °C; (b) 424 ppmv-31.9 °C; (c) 888 ppmv-28.0 °C; and (d) 940 ppmv-31.8 °C. Linear mixed effects modelling across all treatments (Table S4) revealed that reef zone was not a significant ($p > 0.05$) predictor of calcification rate. However, nearshore corals calcified faster ($p < 0.008$) than forereef corals in the high 888 ppm-v/28 °C treatment (Fig. S6). Black vertical bars show standard error.

(a)

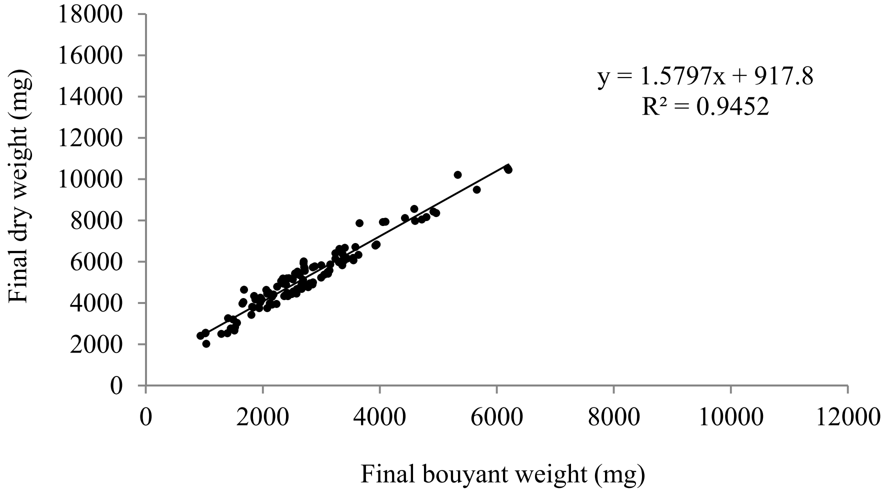

(b)

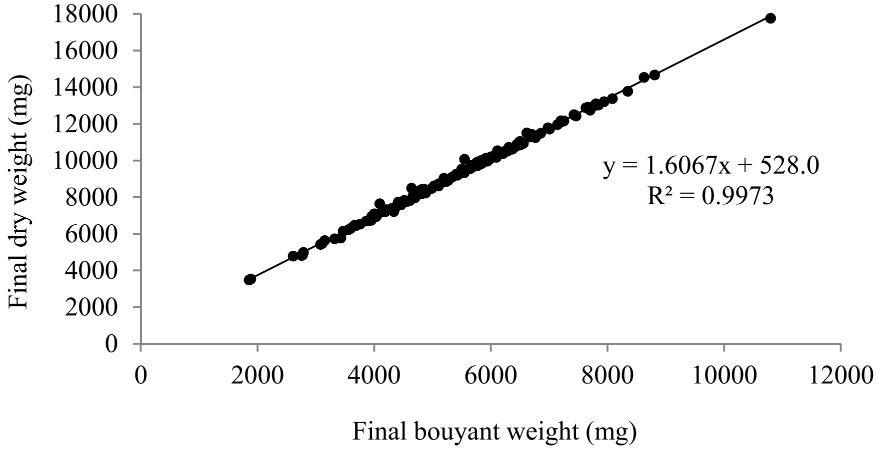

Figure S6. Final buoyant weight vs. final dry weight for *S. siderea* corals in low-pCO_2 (a) and high-pCO_2 (b) treatments, including specimens that died before experiment was concluded. The strong linear correlations allow for estimation of dry weights from measured buoyant weights in determination of net calcification rate.

Table S1. Dry weight, buoyant weight, and surface area data for the *S. siderea* coral specimens investigated in the experiment. Dry weights were calculated using the empirically derived buoyant weight-dry weight regression equations.

Treatment	Replicate	Colony	buoyant weight (mg)			dry weight (mg)			Surface area	Calc. rate normalized to surface area (mg cm^{-2})		
			**0 d**	**30 d**	**60 d**	**0 d**	**30 d**	**60 d**	**(cm^2)**	**0-30 d**	**30-60 d**	**0-60 d**
31.9 °C at 424 ppm-v	**1**	G	2359	2433	2493	4644	4761	4855	2.88	40.43	32.93	73.37
		G	2940	2998	3047	5562	5654	5732	3.39	27.20	22.85	50.05
		H	1637	1669	1677	3504	3554	3567	1.88	26.59	7.00	33.59
		K	993	1012	1017	2486	2516	2525	1.37	21.46	6.52	27.98
		A	2003	2046	2100	4081	4149	4236	3.42	19.85	25.24	45.09
		A	3046	3100	3139	5729	5814	5876	4.18	20.41	14.74	35.15
		B	2316	2375	2398	4576	4670	4706	3.44	27.10	10.57	37.67
		C	1359	1383	1393	3065	3102	3119	2.64	14.17	6.39	20.56
		Q	2617	2651	2663	5052	5106	5124	2.99	18.12	5.98	24.10
		N	1570	1625	1651	3397	3484	3526	2.04	42.57	20.64	63.21
		O	3227	3292	3357	6016	6119	6221	3.77	27.26	27.12	54.38
		P	6088	6141	6184	10535	10619	10687	6.25	13.39	10.86	24.26
	**2**	G	2122	2194	2311	4269	4384	4569	3.14	36.45	59.12	95.57
		G	2853	2914	2997	5425	5522	5652	4.38	22.13	29.83	51.96
		H	1819	1821	1835	3791	3794	3817	2.01	1.57	11.26	12.83
		K	1819	1863	1953	3791	3861	4002	3.13	22.35	45.21	67.56
		A	3529	3578	3652	6493	6569	6687	3.60	21.23	32.80	54.03
		A	1577	1648	1668	3409	3521	3552	1.76	63.76	17.66	81.42
		B	2425	2544	2553	4748	4936	4951	3.01	62.52	5.08	67.59
		C	1749	1801	1818	3681	3763	3790	2.44	33.48	11.02	44.49
		P	4721	4745	4796	8376	8414	8494	4.90	7.74	16.23	23.97
		N	2458	2524	2592	4801	4905	5012	3.01	34.51	35.74	70.24
		O	2488	2636	2716	4848	5082	5208	2.99	78.39	42.28	120.68
		P	2808	2838	2856	5353	5401	5429	3.63	13.21	7.84	21.05
	**3**	G	2368	2397	2413	4658	4705	4730	2.38	19.69	10.62	30.31
		G	2021	2054	2074	4110	4163	4194	2.55	20.89	12.00	32.88

		H	3121	3132	3152	5848	5865	5898	3.20	5.43	10.21	15.64
		K	2129	2163	2181	4280	4335	4363	3.49	15.56	8.16	23.72
		A	4516	4555	4588	8052	8114	8166	5.30	11.71	9.83	21.54
		A	1780	1832	1849	3730	3812	3839	2.40	34.26	11.20	45.45
		A	2607	2667	2687	5036	5131	5162	5.61	17.07	5.53	22.60
		C	2509	2561	2575	4881	4964	4986	3.20	25.85	6.75	32.60
		M	3299	3393	3402	6129	6278	6292	2.69	55.48	5.29	60.77
		N	2204	2304	2342	4399	4557	4617	2.50	63.50	24.05	87.55
		O	3013	3055	3105	5677	5743	5823	4.51	14.70	17.62	32.33
		P	3970	4088	4095	7189	7376	7387	4.15	45.09	2.67	47.75
28.1 °C at 426 ppm-v	**1**	G	1349	1430	1557	3049	3176	3377	2.29	55.37	87.76	143.13
		G	2095	2180	2364	4227	4361	4653	3.79	35.31	77.01	112.32
		H	2548	2612	2696	4943	5045	5176	2.37	42.88	55.55	98.43
		K	917	929	934	2366	2385	2393	1.50	12.67	4.93	17.60
		A	2870	3025	3240	5451	5696	6037	5.18	47.30	65.81	113.10
		A	2389	2442	2445	4692	4775	4780	2.17	38.38	2.43	40.80
		B	1754	1837	1954	3688	3819	4004	2.05	63.83	89.98	153.82
		C	2438	2571	2699	4769	4979	5181	2.48	84.72	81.32	166.04
		M	3353	3421	3549	6215	6322	6525	2.21	48.80	91.41	140.21
		N	2279	2503	2707	4517	4872	5195	3.03	117.17	106.39	223.56
		O	2554	2555	2580	4952	4954	4993	3.22	0.65	12.09	12.74
		P	4622	4628	4716	8220	8229	8368	4.25	2.10	32.68	34.78
	**2**	G	4216	4352	4602	7578	7792	8188	5.30	40.31	74.66	114.96
		G	1935	2044	2234	3975	4147	4446	2.70	63.60	111.01	174.61
		H	1837	1872	2157	3820	3876	4325	2.75	20.12	163.64	183.76
		K	1723	1872	2157	3639	3876	4325	2.80	84.50	160.72	245.22
		A	3275	3399	3544	6091	6287	6516	2.65	73.61	86.50	160.11
		A	1829	1939	2059	3807	3981	4170	2.73	63.75	69.34	133.09
		B	2150	2267	2415	4314	4500	4733	3.05	60.73	76.60	137.34
		C	1787	1921	2074	3741	3952	4194	2.40	88.13	100.40	188.53

		Q	870	948	1034	2292	2416	2551	1.24	99.71	108.62	208.34
		N	2146	2294	2551	4308	4541	4948	3.03	76.94	134.25	211.18
		O	3128	3233	3442	5859	6024	6355	3.84	43.07	86.00	129.07
		P	5449	5477	5659	9525	9570	9857	4.90	9.14	58.71	67.85
	**3**	G	2267	2321	2426	4499	4585	4751	2.90	29.59	57.18	86.76
		G	3853	3902	4052	7004	7082	7318	5.42	14.39	43.56	57.95
		H	2226	2278	2352	4434	4516	4633	2.80	29.51	41.55	71.06
		K	1243	1340	1492	2881	3034	3275	2.38	64.36	101.29	165.64
		A	1220	1237	1289	2845	2871	2954	1.83	14.39	45.18	59.56
		A	1728	1800	1872	3647	3761	3875	2.39	47.53	47.75	95.28
		F	2397	2444	2519	4704	4779	4898	3.74	20.01	31.85	51.87
		C	1414	1437	1450	3151	3188	3208	2.01	18.34	10.22	28.56
		M	2686	2732	2848	5161	5234	5417	3.48	20.87	52.63	73.50
		N	2351	2452	2586	4631	4791	5002	3.08	51.97	68.56	120.53
		O	2383	2513	2630	4682	4887	5072	3.02	68.05	61.24	129.29
		P	3866	3871	3945	7025	7033	7150	3.62	2.04	32.33	34.37
31.8 °C at 940 ppm-v	**1**	A	5469	5518	5546	9315	9394	9439	5.30	14.86	8.49	23.35
		E	5058	5087	5104	8655	8702	8729	6.00	7.77	4.55	12.32
		D	2752	2769	2769	4950	4977	4976	4.55	6.00	-0.12	5.89
		E	3741	3770	3759	6538	6585	6568	5.10	9.23	-3.46	5.77
		G	6309	6354	6367	10664	10737	10758	7.09	10.28	2.95	13.22
		H	4782	4800	4814	8211	8240	8263	6.34	4.56	3.72	8.28
		K	4881	4886	4878	8370	8378	8365	4.70	1.71	-2.85	-1.14
		M	4383	4392	4396	7570	7585	7591	5.03	2.87	1.17	4.04
		P	5188	5216	5232	8864	8909	8934	6.44	6.98	3.91	10.89
		N	5161	5184	5194	8820	8857	8874	6.01	6.24	2.76	9.00
		P	6969	7008	7004	11726	11788	11781	6.37	9.75	-1.09	8.66
		R	5785	5824	5836	9823	9885	9905	7.02	8.78	2.90	11.68
	**2**	C	5051	5098	5093	8643	8719	8710	7.48	10.17	-1.22	8.95
		A	3257	3299	3321	5761	5829	5864	4.46	15.14	7.93	23.07

		E	5202	5264	5266	8887	8986	8989	5.67	17.58	0.57	18.15
		F	4311	4323	4319	7454	7473	7468	5.02	3.84	-1.07	2.78
		G	8632	8767	8805	14397	14613	14676	7.89	27.42	7.87	35.30
		I	3060	3100	3107	5445	5509	5519	4.33	14.85	2.48	17.33
		L	3879	3917	3948	6761	6821	6871	5.64	10.64	8.83	19.47
		M	6681	6761	6762	11263	11391	11393	5.47	23.48	0.20	23.68
		N	4803	4864	4874	8246	8344	8359	6.50	15.08	2.31	17.39
		O	4568	4632	4664	7867	7970	8022	5.01	20.44	10.38	30.82
		P	5764	5841	5859	9788	9913	9941	6.13	20.26	4.63	24.88
		P	6926	6999	7004	11656	11773	11781	6.42	18.18	1.25	19.43
	**3**	A	4079	4129	4159	7082	7162	7210	4.49	17.66	10.74	28.40
		D	5984	6047	6049	10142	10244	10247	6.88	14.79	0.47	15.26
		A	6515	6570	6613	10995	11083	11154	7.84	11.28	8.95	20.23
		F	4052	4092	4093	7038	7103	7105	4.56	14.21	0.47	14.67
		H	6042	6068	6093	10236	10277	10318	5.69	7.16	7.25	14.41
		I	6150	6202	6208	10409	10492	10503	6.48	12.82	1.65	14.47
		K	2689	2739	2752	4848	4929	4949	4.80	16.97	4.13	21.11
		L	4562	4591	4612	7858	7905	7938	5.20	8.96	6.38	15.34
		J	4633	4656	4697	7971	8008	8075	5.57	6.64	12.03	18.66
		O	5199	5299	5331	8881	9042	9093	6.25	25.79	8.22	34.01
		Q	7400	7440	7458	12417	12481	12511	8.06	7.97	3.72	11.69
		R	3575	3662	3681	6271	6412	6443	5.40	26.01	5.76	31.77
28.0 °C at 888 ppm-v	**1**	A	4959	5066	5199	8496	8668	8881	5.57	30.86	38.36	69.23
		C	3440	3523	3601	6055	6188	6314	5.09	26.10	24.84	50.94
		D	3390	3449	3470	5975	6069	6103	5.29	17.72	6.38	24.10
		E	5333	5589	5803	9096	9508	9852	6.37	64.74	53.89	118.63
		G	6884	7180	7424	11589	12065	12457	7.58	62.84	51.74	114.58
		H	5586	5652	5731	9502	9609	9737	5.80	18.38	21.98	40.37
		K	3668	3820	3928	6421	6666	6840	5.44	44.96	31.87	76.83
		L	5456	5649	5842	9294	9605	9914	5.78	53.73	53.55	107.28

	N	3080	3249	3426	5477	5749	6033	4.33	62.86	65.71	128.57
	O	4817	4974	5108	8267	8520	8736	4.90	51.68	43.92	95.60
	P	6087	6319	6557	10307	10681	11063	5.47	68.34	69.81	138.15
	Q	5304	5494	5672	9049	9355	9641	6.17	49.58	46.37	95.95
**2**	A	4457	4595	4729	7690	7911	8126	5.15	43.06	41.61	84.67
	B	4032	4173	4336	7006	7233	7494	3.82	59.54	68.22	127.76
	C	2962	3075	3150	5287	5469	5589	4.31	42.09	27.93	70.02
	F	4581	4693	4774	7888	8068	8198	5.76	31.25	22.51	53.75
	H	5886	5994	6100	9986	10159	10329	7.02	24.71	24.17	48.88
	I	6276	6389	6422	10612	10793	10846	7.01	25.82	7.49	33.31
	L	6266	6557	6853	10596	11063	11539	7.28	64.20	65.30	129.50
	M	3238	3457	3658	5730	6082	6405	5.28	66.55	61.28	127.83
	O	6097	6239	6447	10325	10553	10886	6.45	35.40	51.69	87.09
	N	6007	6390	6733	10179	10795	11345	7.55	81.46	72.88	154.35
	P	6821	7072	7251	11487	11890	12179	6.90	58.37	41.84	100.21
	R	4425	4626	4819	7638	7960	8270	7.36	43.76	42.16	85.92
**3**	D	2994	3029	3079	5339	5394	5474	4.73	11.67	16.99	28.66
	D	4140	4171	4186	7180	7229	7254	5.15	9.57	4.78	14.35
	E	5997	6106	6236	10163	10339	10547	5.87	29.92	35.49	65.41
	F	5764	5853	5914	9788	9931	10029	7.10	20.14	13.81	33.95
	G	5910	6196	6502	10023	10484	10975	6.98	65.96	70.41	136.37
	H	7825	7882	7938	13101	13191	13283	5.86	15.45	15.54	30.99
	K	4635	4770	4854	7976	8192	8326	5.83	37.20	22.96	60.16
	M	6338	6512	6670	10711	10990	11245	7.30	38.28	34.83	73.12
	N	3617	3823	4000	6339	6670	6955	5.93	55.94	47.99	103.93
	O	5501	5651	5778	9366	9607	9812	5.85	41.20	35.06	76.26
	Q	7253	7469	7631	12181	12528	12788	8.20	42.35	31.70	74.05
	R	4105	4162	4207	7124	7215	7287	5.02	18.15	14.41	32.56

Table S2. Corallite height (µm) and percent-infilling ('%-I') data for *S. siderea* specimens (shown by 'ID') after 60 days growth in the experimental treatments. 'Rep' = replicate tank number.

**28.1 °C/426 ppm-v**				**31.9 °C/424 ppm-v**				**28.0 °C/888 ppm-v**				**31.8 °C/940 ppm-v**			
ID	Rep	Height	%-I	ID	Rep	Height	%-I	ID	Rep	Height	%-I	ID	Rep	Height	%-I
A104a	1	1670.3	96	A110a	1	1790.0	87	H7	1	1198.5	89	P12	1	1072.7	
G4b		1409.0		A101a		1053.9	89	A17		1146.1	81	K5		1079.9	
N102		1154.6		P101a		1419.6		K18		974.4	83	M13		886.1	
P102a		970.6	92	O101a		1179.7	89	Q1		1778.5	81	A19		1298.6	88
A105a	2	1298.6	89	G19b		1209.4		P3		1315.8	97	P16		1335.3	88
P105a		1560.5	91	G1a		1072.5	97	G18		1203.5	89	G14		1429.5	84
G12a		1532.9	93	A102a	2	1068.2		O11		1169.0	81	H18		1138.0	89
KBb		1172.6	93	KBa		1172.4	95	M17	2	1483.8		N17		1000.9	
O105a		1573.0	85	P105b		1252.3		F18		1279.2		P10	2	1057.3	85
P102b	3	1403.2	89	O104a		1147.9	94	H12		791.7	86	M3		1140.3	
GS4a		1542.6	85	G4a		1317.0	90	A20		1086.7	82	P8		990.0	82
F16b		1408.7		HDa	3	1352.3	92	P17		1188.9	87	A109b		1500.6	80
N103b		1269.1		A103a		1236.3	90	N11		1034.8		O5		1183.2	86
OS7a		1547.9	97	P101b		1766.5	93	O20		1307.0	83	G15		1094.5	95
M109b		1262.9		O107a		1341.6	89	F15	3	1481.2		F4		1311.9	
AS9a		1410.7	94	KCa		1509.1	94	M4		1216.8		A9	3	1094.1	82
HDb		1312.7						G8		1248.3	87	O18		1134.5	85
KCb		1450.8	94					H5		888.6	85	K13		1176.2	91
								K10		918.0	88	H11		1073.0	83
								N20		932.2		L7		1202.8	
								Q13		1537.0	83	FS5		1223.8	
												A15		1057.4	

Table S3. Summary of the hierarchical linear mixed effects models evaluated in this study ('*' = interactive effect; '+' = additive effect).

Model	Fixed Effects	Random Effects
1	Temperature*pCO$_2$*Reef Zone	Random Slopes and Intercepts for Tank and Colony
2	Temperature*pCO$_2$*Reef Zone	Random Slopes of Tank and Colony and Random Intercept for Colony
3	Temperature*pCO$_2$*Reef Zone	Random Slopes of Tank and Colony and Random Intercept for Tank
4	Temperature*pCO$_2$*Reef Zone	Random Slopes for Tank and Colony
5	Temperature*pCO$_2$	Random Slopes and Intercepts for Tank and Colony
6	Temperature*pCO$_2$	Random Slopes of Tank and Colony and Random Intercept for Colony
7	Temperature*pCO$_2$	Random Slopes of Tank and Colony and Random Intercept for Tank
8	Temperature*pCO$_2$	Random Slopes for Tank and Colony
9	Temperature	Random Slopes and Intercepts for Tank and Colony
10	Temperature	Random Slopes of Tank and Colony and Random Intercept for Colony
11	Temperature	Random Slopes of Tank and Colony and Random Intercept for Tank
12	Temperature	Random Slopes for Tank and Colony
13	Temperature+pCO$_2$	Random Slopes and Intercepts for Tank and Colony
14	Temperature+pCO$_2$	Random Slopes of Tank and Colony and Random Intercept for Colony
15	Temperature+pCO$_2$	Random Slopes of Tank and Colony and Random Intercept for Tank

16	Temperature+pCO_2	Random Slopes for Tank and Colony
17	pCO_2	Random Slopes and Intercepts for Tank and Colony
18	pCO_2	Random Slopes of Tank and Colony and Random Intercept for Colony
19	pCO_2	Random Slopes of Tank and Colony and Random Intercept for Tank
20	pCO_2	Random Slopes for Tank and Colony

Table S4. Summary of p-values and AIC output for each of the 20 hierarchical linear mixed effects models evaluated for 0-60 day calcification rate, 0-30 day calcification rate, 30-60 day calcification rate, corallite height, and corallite infilling. Model in bold contains the greatest number of significant ($p < 0.05$) fixed effects, with random effects assigned by AIC. '*' = interactive effect

**Calcification (0-60 day)**	**Model**	**Temperature**	***p*CO_2**	**Temp******p*CO_2**	**Reef Zone**	**Temp* Reef Zone**	***p*CO_2* Reef Zone**	**Temp******p*CO_2* Reef Zone**	**AIC**
	1	0.09	0.65	0.75	0.51	0.52	0.35	0.38	378.46
	2	0.1	0.67	0.76	0.51	0.53	0.35	0.38	377.20
	3	0.08	0.65	0.75	0.50	0.52	0.34	0.37	377.90
	4	0.09	0.66	0.76	0.5	0.52	0.34	0.37	376.63
	5	0.08	0.63	0.72					289.85
	6	0.09	0.64	0.74					288.57
	7	0.08	0.62	0.72					289.62
	8	0.09	0.63	0.73					288.34
	9	0.001							261.95
	10	0.002							260.64
	11	0.001							261.78
	12	0.002							260.47
	13	0.0006	0.08						272.97
	14	0.0008	0.05						271.74
	15	0.006	0.08						272.75
	**16**	**0.0008**	**0.04**						**271.52**
	17		0.31						280.68
	18		0.22						278.87
	19		0.30						280.47
	20		0.21						278.65

**Calcification (0-30 day)**	**Model**	**Temperature**	***p*CO_2**	**Temp******p*CO_2**	**Reef Zone**	**Temp* Reef Zone**	***p*CO_2* Reef Zone**	**Temp******p*CO_2* Reef Zone**	**AIC**
	1	0.69	0.3	0.26	0.27	0.31	0.15	0.18	388.21
	2	0.69	0.3	0.27	0.27	0.31	0.15	0.18	386.73

	3	0.68	0.3	0.27	0.26	0.29	0.14	0.17	387.61
	4	0.69	0.3	0.27	0.26	0.3	0.14	0.17	386.13
	5	0.67	0.29	0.25					302.50
	6	0.68	0.29	0.26					301.02
	7	0.67	0.29	0.26					302.62
	8	0.67	0.29	0.26					300.62
	9	0.001							271.99
	10	0.001							270.22
	11	0.001							271.59
	**12**	**0.001**							**269.83**
	13	0.001	0.29						286.42
	14	0.002	0.25						284.94
	15	0.001	0.29						285.99
	16	0.002	0.24						284.51
	17		0.5						291.77
	18		0.46						289.98
	19		0.49						291.32
	20		0.45						289.53

**Calcification (30-60 day)**	**Model**	**Temperature**	**$p\mathrm{CO}_2$**	**Temp*$p\mathrm{CO}_2$**	**Reef Zone**	**Temp* Reef Zone**	**$p\mathrm{CO}_2$* Reef Zone**	**Temp*$p\mathrm{CO}_2$* Reef Zone**	**AIC**
	1	0.02	0.2	0.25	0.27	0.33	0.34	0.39	422.63
	2	0.03	0.22	0.27	0.27	0.33	0.34	0.4	421.40
	3	0.02	0.2	0.25	0.26	0.32	0.34	0.39	421.22
	4	0.03	0.21	0.27	0.26	0.32	0.34	0.39	419.99
	5	0.02	0.18	0.23					342.47
	6	0.02	0.19	0.24					341.23
	7	0.02	0.18	0.23					341.60
	8	0.02	0.19	0.24					340.36
	9	0.004							318.18
	10	0.005							317.10

	11	0.003							317.35
	12	0.005							316.28
	13	0.001	0.07						327.80
	14	0.002	0.03						326.50
	15	0.001	0.07						326.94
	**16**	**0.001**	**0.03**						**325.64**
	17		0.24						334.79
	18		0.15						332.96
	19		0.24						333.96
	20		0.14						332.12

**Corallite height**	**Model**	**Temperature**	***p*CO_2**	**Temp*pCO_2**	**Reef Zone**	**Temp* Reef Zone**	***p*CO_2* Reef Zone**	**Temp*pCO_2* Reef Zone**	**AIC**
	1	0.32	0.38	0.49	0.6	0.59	0.43	0.43	1028.43
	2	0.31	0.37	0.48	0.6	0.59	0.42	0.42	1026.43
	3	0.33	0.41	0.52	0.54	0.54	0.38	0.38	1026.92
	4	0.32	0.39	0.51	0.54	0.54	0.37	0.38	1024.92
	5	0.28	0.32	0.43					1038.25
	6	0.27	0.31	0.42					1036.25
	7	0.3	0.34	0.46					1037.04
	8	0.29	0.33	0.45					1035.04
	9	0.4							1038.05
	10	0.4							1036.08
	11	0.39							1036.75
	12	0.39							1034.76
	13	0.28	0.01						1032.67
	14	0.28	0.004						1030.67
	15	0.28	0.01						1031.40
	16	0.28	0.005						1029.40
	17		0.01						1038.82
	18		0.003						1036.82

	19		0.01						1037.52
	**20**		**0.003**						**1035.52**

Corallite infilling	Model	Temperature	pCO_2	Temp*pCO_2	Reef Zone	Temp* Reef Zone	pCO_2* Reef Zone	Temp*pCO_2* Reef Zone	AIC
	1	0.5	0.23	0.35	0.27	0.27	0.33	0.36	-2.16
	2	0.49	0.22	0.34	0.26	0.26	0.32	0.35	-4.16
	3	0.48	0.22	0.34	0.27	0.27	0.34	0.36	-4.15
	4	0.47	0.2	0.33	0.26	0.26	0.32	0.35	-6.15
	5	0.87	0.48	0.69					-121.89
	6	0.86	0.47	0.68					-123.89
	7	0.86	0.48	0.69					-123.89
	8	0.86	0.47	0.68					-125.89
	9	0.8							-147.19
	10	0.79							-149.08
	11	0.8							-149.19
	12	0.79							-151.08
	13	0.53	0.002						-144.66
	14	0.52	0.0006						-146.66
	15	0.52	0.002						-146.66
	16	0.51	0.0005						-148.66
	17		0.002						-156.17
	18		0.0004						-158.17
	19		0.001						-158.17
	**20**		**0.0003**						**-160.17**

Table S5. Summary of statistical parameters for hierarchical linear mixed effects models containing the most significant ($p < 0.05$) fixed effects (with random effects assigned by AIC) for 0-60 calcification rate (temperature and pCO_2), 0-30 day calcification rate (temperature only), 30-60 day calcification rate (temperature and pCO_2), corallite height (pCO_2 only), and corallite infilling (pCO_2 only). Relative variance of the random effects (tank, colony) is proportional to the relative magnitude of their impacts on the dependent variable (calcification rate, corallite height, corallite infilling).

**0-60 day calcification**	**Fixed effects**	**Value**	**SE**	***t*-value**	***p*-value**
	Intercept	9.73	1.63	5.99	
	Temperature	-0.270	0.06	-5.00	0.0008
	pCO_2	-0.000977	0.000420	-2.33	0.04
	**Random effects**	**Variance**	**SD**		
	Colony	0.0000490	0.00700		
	Tank	0.000116	0.0108		

**0-30 day calcification**	**Fixed effects**	**Value**	**SE**	***t*-value**	***p*-value**
	Intercept	6.62	1.28	5.17	
	Temperature	-0.19	0.04	-4.38	0.001
	**Random effects**	**Variance**	**SD**		
	Colony	0.0000658	0.00811		
	Tank	0.0000624	0.00790		

**30-60 day calcification**	**Fixed effects**	**Value**	**SE**	***t*-value**	***p*-value**
	Intercept	12.85	2.40	5.36	
	Temperature	-0.36	0.08	-4.51	0.001
	pCO_2	-0.002	0.001	-2.61	0.03
	**Random effects**	**Variance**	**SD**		
	Colony	0.0000414	0.00643		
	Tank	0.000269	0.0164		

Corallite height	Fixed effects	Value	SE	*t*-value	*p*-value
	Intercept	1511.05	72.39	20.87	
	pCO_2	-0.36	0.09	-3.97	0.003
	**Random effects**	**Variance**	**SD**		
	Colony	4.945	2.224		
	Tank	0.000	0.000		

Corallite infilling	Fixed effects	Value	SE	*t*-value	*p*-value
	Intercept	0.965	0.0161	60.06	
	pCO_2	-0.000119	0.0000210	-5.66	0.0003
	**Random effects**	**Variance**	**SD**		
	Colony	0.000000136	0.000369		
	Tank	0.000	0.000		
